# Genetic polymorphisms of cytochrome P450-1A2 (CYP1A2) among Emiratis

**DOI:** 10.1371/journal.pone.0183424

**Published:** 2017-09-21

**Authors:** Mohammad M. Al-Ahmad, Naheed Amir, Subramanian Dhanasekaran, Anne John, Yousef M. Abdulrazzaq, Bassam R. Ali, Salim M. A. Bastaki

**Affiliations:** 1 Department of Pharmacology, College of Medicine and Health Sciences, UAE University, Al Ain, Abu Dhabi, United Arab Emirates; 2 Department of Pathology, College of Medicine and Health Sciences, UAE University, Al Ain, Abu Dhabi, United Arab Emirates; 3 Department of Pediatrics, College of Medicine and Health Sciences, UAE University, Al Ain, Abu Dhabi, United Arab Emirates; Indiana University, UNITED STATES

## Abstract

Cytochrome P450 1A2 (CYP1A2) is one of the CYP450 mixed-function oxidase system that is of clinical importance due to the large number of drug interactions associated with its induction and inhibition. In addition, significant inter-individual differences in the elimination of drugs metabolized by CYP1A2 enzyme have been observed which are largely due to the highly polymorphic nature of *CYP1A2* gene. However, there are limited studies on CYP1A2 phenotypes and *CYP1A2* genotypes among Emiratis and thus this study was carried out to fill this gap. Five hundred and seventy six non-smoker Emirati subjects were asked to consume a soft drink containing caffeine (a non-toxic and reliable probe for predicting CYP1A2 phenotype) and then provide a buccal swab along with a spot urine sample. Taq-Man Real Time PCR was used to determine the CYP1A2 genotype of each individual. Phenotyping was carried out by analyzing the caffeine metabolites using High Performance Liquid Chromatography (HPLC) analysis. We found that 1.4%, 16.3% and 82.3% of the Emirati subjects were slow, intermediate and rapid CYP1A2 metabolizers, respectively. In addition, we found that 1.4% of the subjects were homozygote for derived alleles while 16.1% were heterozygote and 82.5% were homozygote for the ancestral allele. The genotype frequency of the ancestral allele, CYP1A2*1A/*1A, is the highest in this population, followed by CYP1A2 *1A/*1C and CYP1A2 *1A/*1K genotypes, with frequencies of 0.825, 0.102 and 0.058, respectively. The degree of phenotype/genotype concordance was equal to 81.6%. The CYP1A2*1C/*1C and CYP1A2*3/*3 genotypes showed significantly the lowest enzyme phenotypic activity. The frequency of slow activity CYP1A2 enzyme alleles is very low among Emiratis which correlates with the presence of low frequencies of derived alleles in *CYP1A2* gene.

## Introduction

Cytochrome P450 1A2 (CYP1A2) is one of the CYP450 mixed-function oxidase system that is responsible for the metabolism of xenobiotics in the body and is involved in the synthesis of cholesterol, steroids and other lipids [1]. In addition, CYP1A2 is an important enzyme that bioactivates a number of procarcinogens including polycyclic aromatic hydrocarbons, heterocyclic aromatic amines/amides, mycotoxins and some natural compounds such as aristolochic acids present in several Chinese herbal medicines. Furthermore, this enzyme metabolizes a large number of essential endogenous compounds including retinols, melatonin, steroids, uroporphyrinogen and arachidonic acids [2]. In humans, the CYP1A2 enzyme, encoded by the *CYP1A2* gene, is of important clinical interest due to the large number of drug interactions associated with its induction and inhibition.

The *CYP1A2* gene is found in a cluster with CYP1A1 and CYP1B1 on the long arm region of chromosome 15, q24.1 [3]. The *CYP1A2* gene spans around 7.8 kb and comprises seven exons and six introns, the first exon being a 55-bp-long noncoding exon. CYP1A2 enzyme is a 515-residue protein with a molecular mass of 58294 Da [3]. CYP1A2 is one of the major CYPs expressed in human liver constituting 13–15% of all CYPs while CYP2D6 represents only 2% of the total [4]. Moreover, numerous genomic variations have been reported as the underlying cause of inter-individual differences in CYP1A2 activity [4,5]. In addition, more than 15 and 40-fold variations in mRNA and protein expression levels have been observed in the human liver with a significant number of inhibitors and inducers of expression [6].

However, more than 177 single-nucleotide polymorphisms (SNPs) of human CYP1A2 upstream sequence have been determined “from which 41 variant alleles (*1B to *21) have been published in the Human Cytochrome P450 Allele Nomenclature Committee home page” (www.imm.ki.se/CYPalleles), illustrating the alterations in DNA sequence levels and in some cases their epidemiological frequencies [7]. Twenty-four haplotypes related to changes in the non-coding region and 17 haplotypes related to amino acid changes in the coding region of the CYP1A2 protein have been observed [8].

Additionally, there are over 100 substrates reported for CYP1A2, including many clinically significant drugs like clozapine, tacrine, procarcinogens (e.g. benzopyrene and aflatoxin b1), and endogenous substrates (e.g. steroids and arachidonic acid) [3]. Nevertheless, several studies have implicated *CYP1A2* gene polymorphisms in cancer susceptibility [9,10] and other diseases [11]. Similarly, CYP1A2 variants have been reported in myocardial infarction [12], hypertension [13], Parkinson’s disease [14], schizophrenic patients treated with antipsychotics drug [15], major depressive disorder [16], pancreatic cancer [9], bladder cancer [10] and porphyria cutanea tarda [11]. Huge inter-individual differences in the elimination of drugs that are metabolized by CYP1A2 have been reported, which involves both genetic and environmental factors as the underlying causes [3,17–19].

Furthermore, CYP1A2 variants can also cause alterations in the metabolism of many drugs [7]. To illustrate, CYP1A2 variants might be associated with lowered clearance of theophylline [20], increased toxicity of lidocaine due to clearance inhibition [21], decreased efficiency of antipsychotics like clozapine and olanzapine [22,23,24], increased toxicity of leflunomide in patients with rheumatoid arthritis [25], and alteration of antithrombotic effect of clopidogrel [26].

Limited genomic studies on drug metabolizing enzymes among Emiratis had been conducted including one on NAT2 [27] and CYP2D6 [28] to determine NAT2 and CYP2D6 alleles and their frequencies. The UAE national population is made up of waves of migration from Yemen through Oman as well as Arab migration from central and northern Arabia as well as from Baluchistan [29,30]. In addition, the UAE population consists of immigrants from Iran, Pakistan, Philippines, Bangladesh and Europe [30]. Therefore, this study was carried out to determine CYP1A2 alleles and genotypes, their frequencies and their correlation with the caffeine metabolism phenotypic status.

## Materials and methods

### Subjects

In order to determine *CYP1A2* genotypes and correlations between phenotypes and genotypes in UAE National population, this study was performed on 576 non-smoker Emirati volunteers aged between 16 and 51 years. Though the UAE national population is very heterogeneous, the ethnic group was not considered as a factor in the selection of the sample. A person was considered an Emirati if his father was an UAE citizen. This study is a cross sectional study in which samples were selected in a multi-staged stratified sampling method. Subjects were selected using sampling proportional to size methods in 3 main geographical areas, Abu Dhabi Emirate, Dubai Emirate and the Northern Emirates. The assigned number of individuals who participated from each Emirate was defined based on UAE National Bureau of Statistics of UAE (Emiratis) population from multicenter throughout the UAE. For example since 50% of the Emirati population were based in Abu Dhabi Emirate, we selected 250 subjects (50% of the studied sample) from this Emirate. In the second stage, we collected the list of schools from the different education zones; for example Abu Dhabi Educational Authority provided us with a list of schools within Abu Dhabi Emirate. These totalled 42 schools, situated in 3 zones (Abu Dhabi, Al Ain and Al Gharbia zones). One of our study’s inclusion criteria was that the recruited subjects should be more than 12 years old. For this reason, we selected the schools that had the largest number of Emiratis. We found 4 schools that met this criteria and had a total of more than 450 Emirati students (including all the levels) whereas the remaining schools had ≤ 100 Emirati students in total. The same method was used for the Al Ain zone (we chose 3 schools) and Al Gharbia zone from where we chose 2 schools. We recruited between 27 and 30 participants from each school (9 schools X 30 participants = 270). The same exercise was done in the other Emirates (Dubai = 4 schools and Northern Emirates = 5 schools).

In the third stage we, in cooperation with the school supervisors from these schools, selected all the classes from the first secondary to the third secondary levels. We then labelled the pupils’ names with numbers and using online randomization software generated a set based on numbers. In addition, we asked all the Emirati teachers meeting our study criteria to participate in the research. We gave each of them numbers and included these numbers in the randomization software. Out of the 576 samples, 7 (1%) were Emirati teachers.

The subjects were asked to consume 300 mL of a caffeinated soft drink pepsi cola, which contains 32mg of caffeine and two hours later provided a buccal swab and a mid-stream urine sample (The subjects were not allowed to drink any caffeinated drinks in between). After adjustment of pH to <3.5, aliquots of urine samples were stored at -20°C until analysis. Buccal swabs were collected using Isohelix SK1 Buccal Swabs (source: Cell Projects LTD, Kent, UK) and DNA isolation kit and was performed according to the Isohelix DNA Isolation protocol (ref: http://www.isohelix.com/documentation). Each participant provided written, informed consent prior to sample collection. The study was approved by Al-Ain District Human Research Ethics Committee (AAMD/HREC No: 21M059 (research protocol number)) and funded by the Emirates Foundation for Philanthropy.

### CYP1A2 phenotyping and genotyping

CYP1A2 phenotyping was determined using Caffeine, a non-toxic and reliable predictor of CYP1A2 phenotype as previously described [31,32]. Caffeine metabolites were extracted according to standard protocol using liquid–liquid extraction, high-performance liquid chromatography (HPLC) separation and UV detection (Waters 2998PDA, Waters Corp., Milford, MA, USA) [33]. CYP1A2 phenotype was assigned on the basis of a molar (1,7-dimethylxanthine + 1,7-dimethyluric acid)/1,3,7-trimethylxanthine ((17MX+17MU)/137MX) ratio, which served as quantitative determinant of caffeine 3-demethylation activity (CYP1A2) as outlined previously [31,32]. This ratio typically falls into three phenotypes (slow, intermediate or rapid), with a cut-off value of 2.5 and 6.5 and are in agreement with previous studies [31,32,34].

Genotyping of the *CYP1A2* polymorphisms rs2069514 (G-3860A), rs12720461 (C-729T), rs56276455 (G-2116A), rs72547516 (A-2499T) and rs28399424 (C-5090T) that represented the major SNPs/alterations responsible for the phenotype of the corresponding CYP1A2*1C, CYP1A2*1K, CYP1A2*3, CYP1A2*4 and CYP1A2*6 alleles respectively, were performed using TaqMan Real Time PCR assay (Supplied by Life Technologies “Applied Biosystems”, California, USA). The detailed procedure is available in TaqMan^®^ SNP genotyping assays protocol booklet provided by Applied Biosystems (ThermoFisher Scientific, California, USA). This analysis was run on Applied Biosystems Thermal Cycler QuantStudio 7 Flex (278870068) with MicroAmp^™^ Optical 96-well reaction plate. Reaction volume of 20uL was used for all experiments to have a uniformed DNA concentration in all the samples. Quantification of genomic DNA has been done using NanoDrop Spectrophotometer machine for DNA quantification.

Each Taq-Man genotyping assay contained two primers for amplifying the sequence of interest and two Taq-Man Minor Groove Binder (TaqMan MGB) probes for detecting alleles based on the change in fluorescence of the dyes associated with the probes. More details are shown in S1 Table.

After PCR amplification, an endpoint plate read was performed on a Real Time PCR instrument using the fluorescence measurements (Rn) made during the plate read and the Sequence Detection system (SDS) software (ThermoFisher Scientific, California, USA) plots Rn values based on the fluorescence signals from each well. The plotted fluorescence signals indicated which alleles were in each sample “The SDS software plots showed the results of the allelic discrimination run on a scatter plot of Allele 1 versus Allele 2. Each well of the 96-well plate was represented as an individual point on the plot”.

### Statistical analysis

The haplotype construction and analysis were performed using QuantStudio^™^ 6 and 7 Flex Real Time PCR System Software v1.0 thorough Thermo Fisher Cloud Scientific Analysis, instrument type: QuantStudio 7 Real-Time PCR System (ThermoFisher Scientific, California, USA). Slow and rapid *CYP1A2* genotypes were reported according to the Human CYP-Allele Nomenclature Committee (http://www.cypalleles.ki.se/cyp1a2.htm).

Data entry analysis SPSS (Statistical Package for the Social Sciences) version 19 was used for data entry and analysis (Kruskal-Wallis test, Chi Square test “X^2^”, median values, Frequencies, Percentage, Degree of Freedom “df” and Confident Interval “CI”). A p value of 0.05 was considered statistically significant. The deviation from Hardy-Weinberg Equilibrium was also calculated using Court Lab—Hardy-Weinberg (HW) Calculator—Michael H. (2005—2008).

## Results

Out of 576 non-smoker participants, 290 subjects (50.3%; 282 males and 8 females) were born to consanguineous parents, out of which, 185 (63.7%; 181 males and 4 females) had third degree related parents (first cousins) and 105 (36.3%; 101 males and 4 females) were from the same tribe or had the same family name. Five hundred and nineteen (90%; 498 males and 21 females) were healthy whereas 56 (10% “54 males and 2 females”) had past medical history. Out of those with history of illness 3 subjects (0.6%) had allergic conditions, 4 subjects (0.8%) had cardiovascular disease, 23 subjects (4%) had asthma, 2 subjects (0.4%) had epilepsy, one subject (0.2%) had hypercholesterolemia, one case (0.2%) had vertebral disc disease, 4 subjects (0.8%) had diabetes mellitus, 16 cases (2.9%) had Sickle-cell disorder, one (0.2%) with migraine and (0.2%) had thyroid disease. Fifteen subjects (2.6%) reported hypersensitivity to medications. Out of these, 2 (0.3%) were allergic to antibiotics (not specified by the participants), 2 (0.3%) to ibuprofen and 11 (1.9%) of unknown cause.

### Genotype and haplotype frequencies

We identified six different *CYP1A2* diplotypes. The overall derived alleles’ frequency was 0.095 with allele CYP1A2*1C frequency = 0.0636, allele CYP1A2*1K = 0.0287, allele CYP1A2*3 = 0.0027, allele CYP1A2*4 = 0 and allele CYP1A2*6 = 0 while the ancestral allele frequency was found to be 0.905. The most common genotype frequencies found were the ancestral alleles CYP1A2*1A/*1A, followed by CYP1A2*1A/*1C heterozygotes and CYP1A2*1A/*1K heterozygotes with frequencies of 0.825, 0.102 and 0.058, respectively (Table 1).

**Table 1:** The *CYP1A2* genotype frequencies and distribution among Emiratis.

*CYP1A2* genotype	N	Frequency
CYP1A2*1A/*1A	475	0.825
CYP1A2*1A/*1C	59	0.102
CYP1A2*1A/*1K	33	0.058
CYP1A2*1A/*3	1	0.002
CYP1A2*1C/*1C	7	0.012
CYP1A2*3/*3	1	0.002

The characteristic features and frequencies of the various *CYP1A2* haplotypes identified among Emiratis, determined from TaqMan Real Time PCR analysis of genomic DNA are shown in Table 2. The most common haplotypes by far among this population sample belonged to the CYP1A2*1A (rs2069514) gene and occurred with a frequency of 0.905. We found three different *CYP1A2* derived alleles associated with slow activity. The frequencies of these slow alleles CYP1A2*1C, CYP1A2*1K and CYP1A2*3 were less common than the ancestral allele. The allele belonging to the CYP1A2*1C (rs12720461) gene occurred with a frequency of 0.0636. Whereas, the frequency of CYP1A2*1K (rs56276455) and CYP1A2*3 (rs72547516) alleles were negligible and occurred with a frequency of 0.0287 and 0.0027, respectively. Alleles CYP1A2*4 (rs28399424) and CYP1A2*6 (rs2069514) were absent in this population (Table 2).

**Table 2:** The characteristic features and frequencies of the various *CYP1A2* haplotypes identified among Emiratis.

Haplotype	dbSNP	Enzyme activity	N	Percentage	Hardy-Weinberg Equilibrium
*1A	rs2069514	Normal	1043	90.5%	
*1C	rs12720461	Decreased	73	6.36%	Var. allele freq. = 0.07, P = 0.001
*1K	rs56276455	Decreased	33	2.87%	Var. allele freq. = 0.03, P = 0.44
*3	rs72547516	Decreased	3	0.27%	Can’t be counted●
*4	rs28399424	Decreased	0	0%	Can’t be counted●
*6	rs2069514	Decreased	0	0%	Can’t be counted●

Var. allele freq. = Variant allele frequency

^●^Not accurate if <5 individuals in any genotype group.

While examining the deviation from Hardy-Weinberg equilibrium, it was also found that the observed genotype frequencies were different from the expected ones. Nonetheless, the data for CYP1A2*1K were in accordance with the Hardy-Weinberg equilibrium since P>0.05. For CYP1A2*1C, the data did not obey the equilibrium since P = 0.001. CYP1A2*3, CYP1A2*4 and CYP1A2*6 were too rare for the equilibrium to be determined (Table 2).

**Fig 1:**
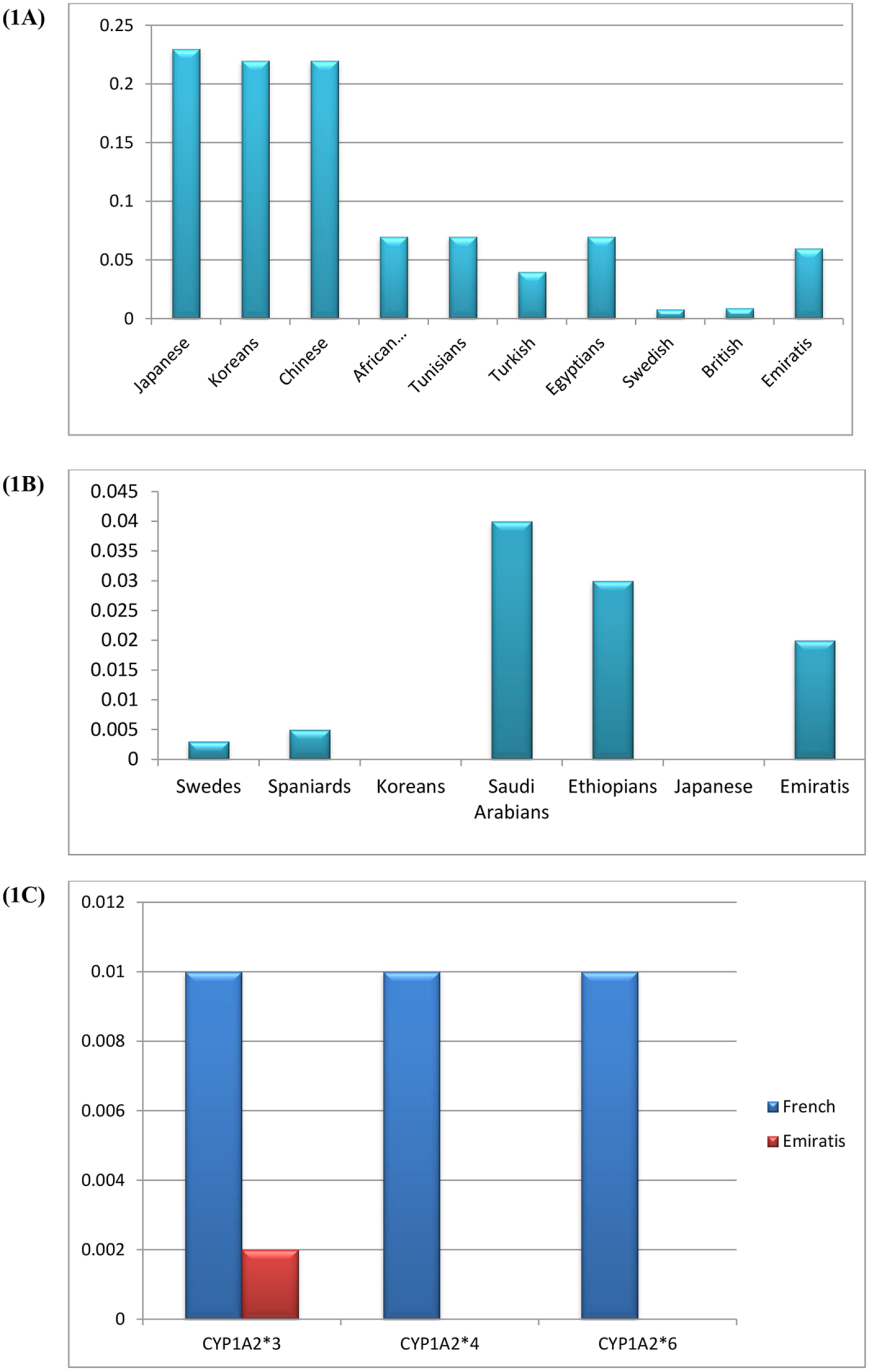
The frequency of *CYP1A2* alleles among Emiratis in comparison with other populations. Fig (1A) shows the CYP1A2*1C haplotype frequencies, Fig (1B) shows the CYP1A2*1K haplotype frequencies, Fig (1C) shows the CYP1A2*3, CYP1A2*4 and CYP1A2*6 haplotypes frequencies, n = sample size, Emiratis (n = 576), Japanese (n = 250), Koreans (n = 150), Chinese (n = 168), African Americans (n = 112), Tunisians (n = 98), Turkish (n = 110), Egyptians (n = 212), Swedish (n = 194), French (n = 100), British (n = 114), Spaniards (n = 117), Saudi Arabians (n = 136), Ethiopians (n = 173).

Based on our study, the Emirati population had the lowest frequencies of CYP1A2*1C of 0.06 when compared to 0.23 in Japanese, 0.26 in Korean, 0.22 in Chinese, 0.07 in African American, 0.07 in Tunisian, and 0.07 in Egyptian populations but higher than in the British (0.009), Swedish (0.008) and Turkish (0.04). The number of the subjects used in our study was higher when compared to other studies (Fig 1A).

The genotype frequency of CYP1A2*1K haplotype was 0.02 in Emiratis populations which was slightly lower when compared to 0.003 in Swedish, 0.005 in Spanish, 0.04 in Saudi Arabian and 0.03 in Ethiopian populations, whereas it was absent in Korean and Japanese populations (Fig 1B).

The genotype frequencies of CYP1A2*3, CYP1A2*4 and CYP1A2*6 alleles were found to be 0.002. The frequencies of CYP1A2*4 and CYP1A2*6 haplotypes were absent in Emiratis population (Fig 1C).

### Phenotype-genotype relationships

The caffeine phenotyping test for CYP1A2 was performed on 560 subjects as there were data missing for 16 (2%) of the subjects out of 576 individuals recruited for the study. Table 3 shows enzyme capacity of six subgroups assigned to *CYP1A2* allele combinations. The CYP1A2*1C/*1C and CYP1A2*3/*3 genotypes showed the lowest median values 2.06 and 2.00 respectively.

**Table 3:** (17MX + 17MU)/137MX molar ratios in subgroups assigned to *CYP1A2* allele combinations.

*CYP1A2* phenotype	*CYP1A2* genotype	N	Frequency	Median	Minimum	Maximum
**Rapid**	CYP1A2*1A/*1A	461	0.825	7.58	6.08	25.25
**Intermediate**	CYP1A2*1A/*1C	57	0.102	4.98	3.13	6.96
	CYP1A2*1A/*1K	33	0.058	4.87	2.53	6.87
	CYP1A2*1A/*3	1	0.002	6.85	6.85	6.85
**Slow**	CYP1A2*1C/*1C	7	0.012	2.06	0.1	2.39
	CYP1A2*3/*3	1	0.002	2.00	2.00	2.00
	**Total**	560				

The resulting urinary (17MX + 17MU)/137MX ratios and genotypic assignments of slow (S/S homozygotes), intermediate (R/S heterozygotes) and rapid activity (R/R homozygotes) are presented in Table 4. Using Kruskal-Wallis test, the mean rank of these groups were 4.5, 67.71 and 327.29 respectively, which showed a strong association between *CYP1A2* genotype and phenotype (X^2^ = 219.23, df = 2 and P value < 0.0001; 95% CI of differences).

**Table 4:** (17MX + 17MU)/137MX ratios vs genotypes.

	S/S homozygotes	R/S heterozygotes	R/R homozygotes
N	8	91	461
Mean	1.8400	5.0830	9.3085
Median	2.0300	4.9800	7.5800
Std. Deviation	0.74310	1.36216	3.37762
Range	2.29	3.95	18.75
Minimum	0.10	2.53	6.08
Maximum	2.39	6.98	25.25

S/S homozygotes = slow metabolizers, R/S heterozygotes = intermediate metabolizers, R/R homozygotes = rapid metabolizers

There was clear evidence of a tri-modal distribution of (17MX + 17MU)/137MX ratio with an apparent anti-mode in the region of 2.5 and 6.5

**Fig 2:**
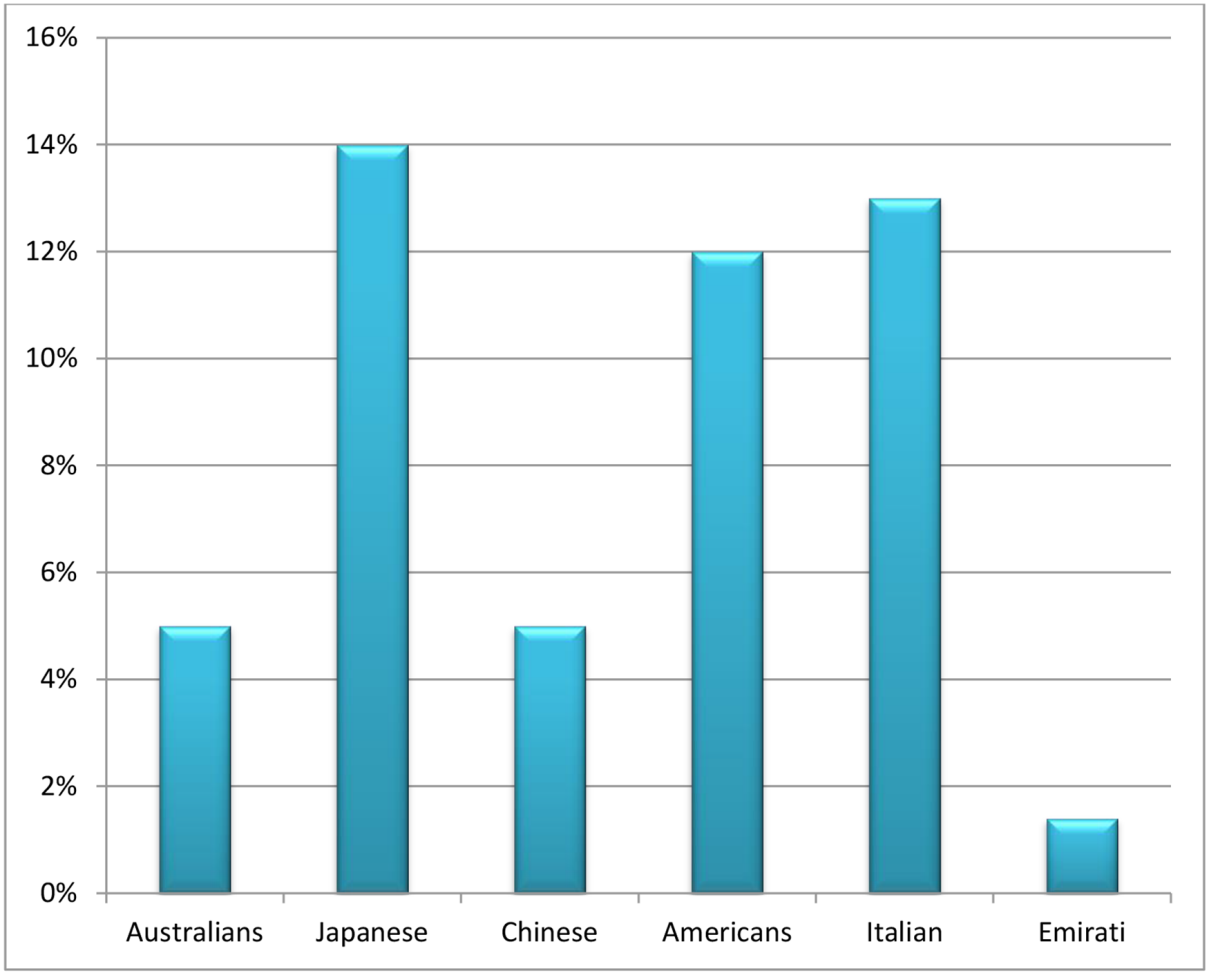
The frequency of CYP1A2 poor metabolizers among Emiratis in comparison with other populations. n = sample size, Emiratis (n = 576), Australians (n = 171), Japanese (n = 250), Chinese (n = 78), Americans (n = 101), Italians (n = 95). We did not compare levels of metabolites or their ratios in different studies, but compared rates of ‘poor metabolizers’ or ‘rapid metabolizers’, whatever cut-off points were used in different studies.

In this study, the phenotype status of CYP1A2 activity in the Emirati population showed the lowest frequency of poor metabolizers with only 1.4% slow phenotype while the percentage in other populations such as Australians, Japanese, Chinese, Americans and Italian were 5%, 14%, 5%, 12% and 13% respectively (Fig 2).

## Discussion

This is the first study to determine the *CYP1A2* genotypes and phenotypes in UAE population, in which certain mutations or polymorphisms of this gene have been genotyped. Sample size selection was made according to the distribution of Emirati population in each Emirate and based on the data provided by UAE National Bureau of Statistics for Emiratis, thus representing the whole UAE Emirati population.

Generally, the exact positioning of the cut-off point (antimode) between phenotypically slow and rapid activities is laboratory-dependent and attributed to differences in chromatographic conditions [35, 36]. As can be seen from the results, there was a clear evidence of a tri-modal distribution of (17MX + 17MU)/137MX ratio with an apparent anti-mode in the region of 2.5 and 6.5 which were in close agreement with that observed in several studies [37]. The mode corresponding to the rapid enzyme activity was not uniformly distributed and may include both heterozygous and homozygous rapid phenotype. The frequency distribution suggested the possible existence of a second anti-mode (in the region of 6.5) separated the heterozygous and homozygous rapid phenotype. The degree of phenotype/genotype concordance was equal to 81.6% based on the current phenotype classification (i.e. rapid/intermediate/slow phenotypes), where those who are homozygotes for the ancestral allele CYP1A2*1A genotypes are considered to be rapid metabolizers, those who are heterozygotes for the ancestral allele are considered to be intermediate metabolizers and those who have no CYP1A2*1A genotype are considered to be slow metabolizers.

CYP1A2*1A has been the ancestral allele for the gene that is associated with rapid phenotype status and based on many studies in many ethnic groups, allele *1A was the most common allele [7,8]. Derived alleles CYP1A2*1C “G-3860A (rs2069514)”, CYP1A2*1K “T-739G (rs2069526), C-729T (rs12720461) and C-163A (rs762551)”, CYP1A2*3 “G-2116A and T-5347C (rs56276455)”, CYP1A2*4 “A-2499T (rs72547516)” and CYP1A2*6 “C-5090T (rs28399424)” had low catalytic activity associated with the slow phenotype [7,8] and were less common than the ancestral allele [38, 39].

In our study G-3860A, C-729T, G-2116A, A-2499T and C-5090T SNPs were investigated and we reported the presence of G-3860A, C-729T and G-2116A variants only, while the A-2499T and C-5090T variants were absent in this population. The distribution of *CYP1A2* gene polymorphisms in our studied population showed a low percentage of derived alleles (9.5%) and a high percentage of the ancestral allele (90.5%).

As a matter of fact, some reports suggested that many influential factors play important roles in correlating *CYP1A2* genotype to CYP1A2 phenotype [3,17–19]. For instance, the drugs which cause induction or inhibition of CYP1A2 enzyme may affect the correlation between *CYP1A2* genotype and phenotype. Similarly, environmental factors such as cigarette smoking can affect CYP1A2 enzymatic activity as well [40]. With regards to our study, all of the participants were non-smokers and the majority of them were aged between 16–19 years of age. Additionally, 90% of them were with no significant past medical history and were not on any concomitant drugs. Thus, we found the high degree of concordance between phenotype and genotypes of *CYP1A2* gene. Interestingly, we found that those with derived alleles had shown slow phenotype status. Furthermore, those who were homozygote for alleles CYP1A2*1C and CYP1A2*3 were associated with the slowest enzyme activity. We did not compare levels of metabolites or their ratios in different studies, but compared rates of ‘poor metabolizers’ or ‘rapid metabolizers’, whatever cut-off points were used in different studies.

Hence, when Hardy-Weinberg equation was applied, the apparent numbers of homozygous and heterozygous slow activity were not in strict accordance with the Hardy-Weinberg equilibrium which is a clear indication that individual genotypes cannot be predicted. The Hardy Weinberg equation is an equation used to calculate the genetic variation in a population at equilibrium. The Hardy Weinberg equilibrium states that the amount of variation in a population remains constant from one generation to the next if there are no perturbing factors. This occurs when mating is random in a large population.in which both the frequencies of genotypes and alleles remain constant because they are in equilibrium. This equilibrium can be disturbed by many factors like mutations, natural selection, non-random mating and genetic drift. For allele CYP1A2*1C the result was inconsistent with Hardy-Weinberg Equilibrium which may be due to an unexpectedly large number of CYP1A2*1C homozygotes which is again related to high level of consanguinity in the population. For allele CYP1A2*1K the result was consistent with Hardy-Weinberg Equilibrium. This finding may be due to the evolutionary influences, like mate choice, mutation, selection, genetic drift, gene flow and meiotic drive as one or more of these influences are typically present in real populations. The level of consanguinity in the UAE is very high with more than 50% of marriages being consanguineous [41]. Indeed, about 50% of the sample group reported consanguinity of their parents. This clearly influences the genotype frequencies and their deviation from Hardy-Weinberg. Another possibility is genotyping error.

Based on epidemiological studies, it has been clearly observed that there is a significant ethnic variability in the distribution of common and rare *CYP1A2* SNPs and haplotypes [8]. The Emirati population had the lowest frequencies CYP1A2*1C when compared to the Japanese, Korean, Chinese, African Americans, Tunisian, and Egyptian but were higher than in British, Swedish and Turkish [23, 42–45]. Furthermore, the frequency of CYP1A2*1K haplotype in this Emirati population was slightly lower than in the Swedish, Spanish, Saudi Arabian and Ethiopian populations, whereas it was absent in Korean and Japanese populations [46, 47]. The genotype frequencies of CYP1A2*3, CYP1A2*4 and CYP1A2*6 alleles that have been identified in the French population [8,31, 36] were found to be lower in the Emirati population specifically the haplotype CYP1A2*3 frequency. Similarly, the frequencies of CYP1A2*4 and CYP1A2*6 haplotypes were absent in Emiratis population. An obvious choice for studying genotype and phenotype concordance would have been CYP1A2*1F as it is one of the most commonly found polymorphisms. We did not study CYP1A2*1F because it represents a highly inducible genotype associated with increased CYP1A2 activity upon exposure to certain inducing agents like caffeine. It changes its activity depending on the drug consumed, for example smoking induces it greatly. It also represents “slow metabolizers” of caffeine.

Study limitations: Firstly, though the UAE national population is very heterogeneous and is made up of waves of migration from Yemen through Oman as well as Arab migration from central and northern Arabia, the ethnic group was not considered as a factor in the selection of the sample. A person was considered an Emirati if his father was an UAE citizen and thus exclusion of genetic diversity. Secondly, the laboratory techniques can differ from laboratory to laboratory and thirdly, our approach does not allow for the identification of novel and other rare variants.

In conclusion, the frequency of slow activity CYP1A2 enzyme alleles is very low amongst Emiratis which correlates with the presence of low frequencies of derived alleles in *CYP1A2* gene. The genotype frequency of the ancestral allele is the highest in this population, followed by CYP1A2*1A/*1C and CYP1A2*1A/*1K genotypes, respectively.

## Supporting information

S1 Table Assay ID or design that has been used for each SN. (DOCX)
