# Supplementary material for: Genetic polymorphisms of cytochrome P450-1A2 (CYP1A2) among Emiratis
Source: PLoS One. 2017 Sep 21;12(9):e0183424. doi: 10.1371/journal.pone.0183424 (PMC5608188; doi:10.1371/journal.pone.0183424)
Supplement: S1 Table — (DOCX) [file pone.0183424.s001.docx]

**Supplemental Data Table 1:** Assay ID or design that has been used for each SNP

| Assay ID | dbSNP | Haplotype | Forward and Reverse Primers  Context Sequence [VIC/FAM]* | VIC dye-fluorescence signal generated | FAM dye-fluorescence signal generated | Allele Nomenclature |
| --- | --- | --- | --- | --- | --- | --- |
| C__15859191_30 | rs2069514 | At position 3860 | TGGCTCACCGCAACCTCCGCCTCTC[G/A]GATTCAAGCAATTGTCATGCCCCAG | **G** | ● | Ancestral allele |
|  |  |  |  | ● | **A** | Derived allele *CYP1A2*1C* |
| C__30634146_10 | rs12720461 | At position 729 | GGCTAGGTGTAGGGGTCCTGAGTTC[C/T]GGGCTTTGCTACCCAGCTCTTGACT | **C** | ● | Ancestral allele |
|  |  |  |  | ● | **T** | Derived allele *CYP1A2*1K* |
| C__30634247_20 | rs56276455 | At position 2116 | GTACATGGGGGCCCCCAACCCTATA[G/A]ACAGAGGAAGATCCAGAAGGAGCTG | **G** | ● | Ancestral allele |
|  |  |  |  | ● | **A** | Derived allele *CYP1A2*3* |
| C__30634246_10 | rs72547516 | At position 2499 | ACACTCCTCCTTCTTGCCCTTCACC[A/T]TCCCCCACAGGTGAGGCCTGCCGGT | **A** | ● | Ancestral allele |
|  |  |  |  | ● | **T** | Derived allele *CYP1A2*4* |
| C__30634244_20 | rs28399424 | At position 5090 | GGACCCCTCTGAGTTCCGGCCTGAG[C/T]GGTTCCTCACCGCCGATGGCACTGC | **C** | ● | Ancestral allele |
|  |  |  |  | ● | **T** | Derived allele *CYP1A2*6* |

● = No dye-fluorescence signal generated.

*The TaqMan® MGB Probes consisted of target-specific oligonucleotides with: first, a reporter dye was at the 5´ end of each probe (VIC dye was linked to the 5´ end of the Allele 1 probe and FAM dye was linked to the 5´ end of the Allele 2 probe) (Afonina I et al 1997, Kutyavin IV et al 1997). Each Taq-Man MGB Probe annealed specifically to its complementary sequence, if present, between the forward and reverse primer sites. When the probe was intact, the proximity of the reporter dye to the quencher dye resulted in quenching of the reporter fluorescence primarily by Forster-type energy transfer (Lakowicz JR 1983). The primers bounded to the template were extended by AmpliTaq Gold DNA Polymerase which in turn cleaved the probes only that were hybridized to the target. The cleavage separated the reporter dye from the quencher dye, which resulted in increased fluorescence signal by the reporter. Thus, the fluorescence signal generated by PCR amplification indicated which alleles were presented in the sample.
